# A unique case of coexisting intrauterine and abdominal pregnancy which progress to term with a positive birth outcome

**DOI:** 10.1186/s12884-022-04561-x

**Published:** 2022-03-24

**Authors:** Tadiwos Utalo, Jenenu Getu

**Affiliations:** Bele Primary Hospital, Wolaita Zone, Wolaita Sodo, Southern Ethiopia Ethiopia

**Keywords:** Heterotopic pregnancy, Ectopic pregnancy, Abdominal pregnancy, Spontaneous heterotopic pregnancy

## Abstract

**Background:**

The term heterotopic pregnancy is defined as a uterine pregnancy coexisting with a second pregnancy in an extrauterine location. Spontaneous, full-term heterotopic pregnancy with alive birth is very rare. The diagnosis and management of such exceptionally unique case is difficult. When the patient presented with an advanced labor with no antenatal care follow up and with no risk factors is even more challenging for poorly equipped facilities like ours.

**Case presentation:**

A 25 years old gravida 3, para 2 (both are alive) mother presented to the labor and delivery ward of Bele Primary Hospital, Southern Ethiopia with the complaint of pushing down pain of 18 h duration. Immediately after arrival, she gave birth to a 3300gm female neonate spontaneously. After delivery, an abdominal mass was recognized and manual exploration of the uterus was done to look for the presence of after coming second twin but the uterus was empty. On ultrasound examination, there was an alive fetus in transverse lie outside the uterus. With the impression of 2nd twin in a separate horn of bicornuate uterus and to rule out abdominal pregnancy, laparotomy was done. On laparotomy, there was abdominal pregnancy in the Pouch of Douglas with an intact amniotic sac. The sac was attached with the left broad ligament, left ovary, small bowel mesentery, and posterior wall of the uterus. The sac opened, a 1600gm alive female neonate with features of fetal growth restriction and left club foot was delivered. The placenta was detached spontaneously and removed without any complication.

**Conclusions:**

The coexistence of spontaneous full-term intrauterine with advanced abdominal ectopic pregnancy is one of the rarest forms of heterotopic pregnancy. Every health professional should bear in mind that intrauterine and extrauterine pregnancy may happen simultaneously and it can progress to term without any symptoms. Ultrasound is the diagnostic method of choice but the existence of an intra-uterine pregnancy cannot rule out ectopic pregnancy. The life-threatening complication of abdominal ectopic pregnancy is bleeding from the detached placental site. Therefore, the decision to remove the placenta should be individualized.

## Background

The word heterotopic pregnancy is used in place of the older term combined pregnancy [1]. It is defined as simultaneous coexistence of an intra-uterine pregnancy (IUP) and an extrauterine pregnancy [2]. Because, strictly speaking, heterotopic is synonymous with ectopic, the use of "heterotopic ectopic pregnancy" is tautological [1].The fallopian tube is the commonest site of the ectopic implantation in heterotopic pregnancies, but the cervix or abdomen can also be involved [2]. In spontaneous conceptions heterotopic pregnancy is rare and it is estimated to occur in about 1 per 30,000 spontaneous pregnancies [3]. A higher prevalence of heterotopic pregnancy occur in assisted reproduction techniques that may reach up to 1 case per 100 [3]. Majority (about 80%) of heterotopic pregnancies end during the first trimester, so advanced pregnancies are extremely rare [4]. According to literature, there are a few reports of exceptional cases of heterotopic pregnancy that are carried to term with good perinatal outcome for both the intrauterine and extrauterine fetuses [4–6].

Risk factors for the development heterotopic pregnancy are any event that can lead to scarring of the fallopian tube [7]. Risk factors that can increase the risk of heterotopic pregnancy include pelvic inflammatory disease (PID), tubo-ovarian abscess (TOA), previous ectopic pregnancies, or previous pelvic surgery [8]. Heterotopic pregnancy is thought to occur because of multiple ovulation events [9]. Therefore, people who have undergone assisted reproduction therapies are at an increased risk of heterotopic pregnancy [10]. Symptoms of heterotopic pregnancy include abdominal mass, abdominal pain, peritoneal irritation, and enlarged uterus [11]. In some cases there may be either hypovolemic shock or a complete lack of symptoms [12]. Early symptoms can also be similar to those seen in acute appendicitis, ovarian cyst rupture, or ovarian torsion, which makes it more difficult to diagnose [12].The diagnosis of heterotopic pregnancy is challenging, because it is often difficult to identify both the intra-uterine and extra-uterine pregnancy [13]. Ultrasonography is the diagnostic method of choice in detecting heterotopic pregnancy [3]. Magnetic resonance imaging (MRI) [14], and computed tomography (CT) scan [15] are also helpful in equivocal cases to establish the diagnosis and localize the placenta’s implantation site.

Because of the rare occurrence of heterotopic pregnancy, there is little agreement on the optimal surgical management [16]. Treatment of heterotopic pregnancy should be as minimally invasive as possible to preserve the developing intra-uterine pregnancy (IUP) [16].The mainstay of heterotopic pregnancy treatment is surgical, either laparotomy or laparoscopic [3]. Nonsurgical management of the early ectopic pregnancy (EP) was also described in some reports [17]. Treatment of unusual cases of heterotopic pregnancies such as cornual, abdominal, and cervical implantations probably should be individualized [18]. Successful treatment in these situations depends largely on the gestational week of diagnosis, the patient's clinical condition, and the findings in the surgical procedure [3].

Here we present an exceptional case of spontaneous heterotopic pregnancy (intrauterine and abdominal ectopic) which progresses to full term with good perinatal outcome for both the intrauterine and extrauterine fetuses.

## Case presentation

A 25 years old gravida 3, and para 2 (both are alive) mother presented with the complaint of advanced labor pain of 18 h duration. She came by ambulance transport from a 35 km distant rural health center to Bele Primary Hospital, Wolaita Zone, Southern Ethiopia. The mother did not remember her last normal menstrual period but claims to be amenorrheic for the last 9 months. During the current pregnancy; she had no antenatal care visit, no history of vaginal bleeding, no abdominal pain, and no other danger signs of pregnancy. She has no previous history of pelvic inflammatory disease (PID), and pelvic surgery. She has also no history of contraceptive use. Both her last deliveries were at home with no complications. During the physical examination, her vital signs were in the normal range. Pink conjunctiva and non-icteric sclera. On abdominal examination, 38 weeks sized uterus, fetal heart beat was 148 bpm, cephalic presentation, longitudinal lie, multiple fetal poles were not appreciated, there was 3 uterine contractions in 10 min with moderate strength and bladder was not distended. On the genito-urinary examination (per vagina), cervix was fully dilated, vertex presentation, fetal head visible at vulva, normal position, no sign of caput or molding, the membrane was ruptured with clean amniotic fluid.

Basic laboratory investigations were done, her hematocrit level was 35%, and her blood group was “O positive”. Other serologic tests were also done for HIV, Hepatitis, and syphilis and all were negative and urine analysis was also negative for microscope exam.

Vaginal delivery summary, this mother gave birth to alive female neonate weighing 3300gm with an Apgar score of 8 and 9 in the 1^st^ and 5^th^ minutes respectively by spontaneous vaginal delivery and 3^rd^ stage of labor managed actively.

**Fig. 1:**
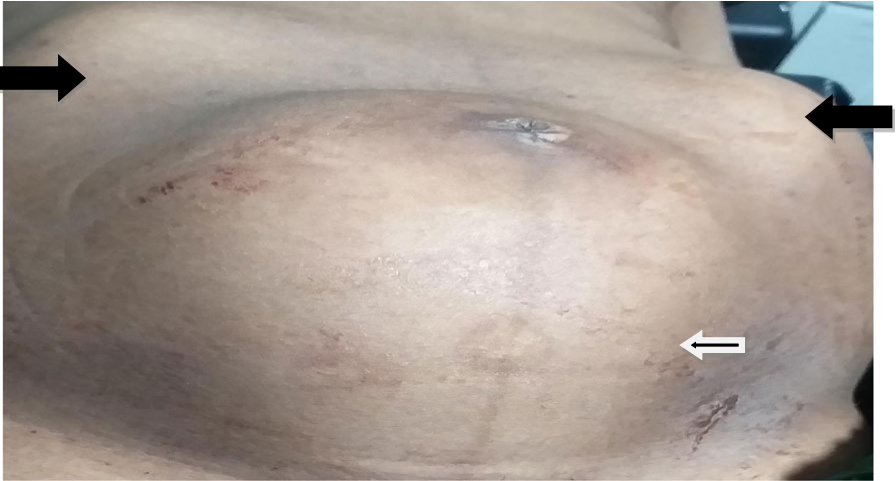
Appearance of the abdomen after delivery of the IUP (white outline arrow- the contracted, empty uterus after delivery, black arrows- the abdominal pregnancy in transverse lie)

But after the delivery of the neonate, her abdomen shows three tumor features i.e. contracted 20 weeks sized uterus and palpable masses at both left and right upper quadrants. The mass was non-tender and slightly hard and smooth at the left side posterior to the uterus and irregular at the right side (Fig. 1). Bimanual exploration of the uterus was made to look for after coming 2^nd^ twin and speculum examination also performed to explore the presence of additional cervical canal and double uterus, but only one cervical opening was appreciated. The posterior fornix was bulged.

On ultrasound examination, a fetus was seen in an intact amniotic sac with scanty fluid posterior to the empty uterus. The fetus was in a transverse lie, the head and placenta were at the left upper quadrant below the spleen and its abdomen and extremities were towards the right upper quadrant of her abdomen. The fetal heartbeat was normal and no gross congenital anomaly was identified.

Referral was planned for the impression of 2^nd^ twin in a separate horn of the bicornuate uterus and to rule out abdominal pregnancy. But due to economic reason, the patient refused referral. Then after getting informed, written consent, and preparing two units of cross-matched whole blood, the patient was taken to OR. Abdomen was entered through a midline vertical skin incision.

**Fig. 2:**
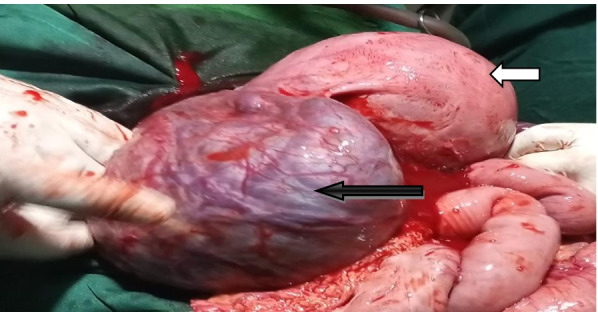
the appearance of the intact amniotic sac and the uterus immediately on opening the abdomen (Dark arrow- intact amniotic sac, white arrow- uterus)

**Fig. 3:**
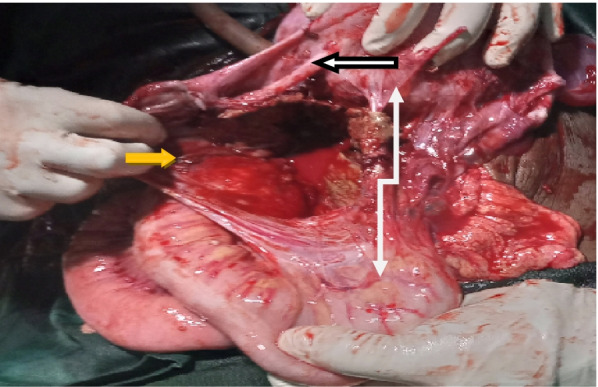
Attachments of the sac to left adnexa (black outline arrow), small bowel mesentery, and posterior wall of the uterus (long white arrow) and placenta separation site (orange arrow)

The intra-Op findings were: A fetus in a transverse lie was found, in its intact amniotic sac posterior to the uterus, in the pouch of Douglas (Fig. 2). The sac was attached with the left broad ligament, left ovary, small bowel mesentery, and posterior wall of the uterus (Fig. 3). The head and placenta were in the left iliac fossa with engorged and tortious vessels which start to rupture spontaneously during manipulation (Fig. 2). Then the intact amniotic sac was opened to deliver alive female neonate with left club foot weighing 1600gm with Apgar score of 7and 8 in the 1^st^ and 5^th^ minutes respectively. The placenta was delivered spontaneously without resistance from its site of attachment. Small bleeders from the placenta detachment site were controlled by multiple ligations. The normal anatomy of the left adnexa was distorted and it was difficult to identify the ovary (Fig. 3). But the right tube and ovary were normal. Fresh edges of the sac sutured and left in place, hemostasis was secured and the abdomen was closed in layers. The patient was transferred to post anesthesia care unit with the post-operative diagnosis of spontaneous full-term heterotopic pregnancy. Her post-Op hematocrit (HCT) level was 29% and the post-operative course was uneventful. Unfortunately, on the 7^th^ post-Op day, the very low birth weight baby died while she was on treatment at the neonatal care unit of our hospital. The patient was discharged home on the 8^th^post-operative day. She returned on her 45^th^ day for post-natal care follow up and both the mother and her baby were in good condition.

## Discussion and conclusions

Heterotopic pregnancies in natural conception are a very rare event [4]. The first case was reported in 1708 as an autopsy finding [19] and this condition is estimated to be less frequent than one in 30,000 spontaneous pregnancies [3]. Assisted reproductive procedures like In-Vitro fertilization (IVF) and induction of ovulation are highly contributing to the occurrence of heterotopic pregnancy [3]. In the National ART Surveillance System out of 553,577 pregnancies conceived by ART between 2001 and 2011, only 485 heterotopic pregnancies were identified–that is 1 per 1111 [20]. Our patient had a spontaneous conception.

Majority of heterotopic pregnancies are diagnosed during the first and early second trimester pregnancies [3, 18, 21, 22]. According to a review done by Barrenetxea et al. of published case reports from January 1994 to December 2004, out of 13 spontaneous heterotopic pregnancies, 74% of them are diagnosed early (between 5 and 8 weeks), the latest being at 20 weeks [21]. Based on a similar review done by Kajdy et al., out of 14 spontaneous heterotopic pregnancies most were diagnosed between 6 and 8 weeks and one was diagnosed at 26 weeks [18]. A systematic review of 18 spontaneous heterotopic pregnancies done by Oancea et al. also revealed that the diagnosis of most heterotopic pregnancies was mainly established in the first trimester, the mean gestational age being 8 weeks [22]. They found only one article describing a diagnosis of heterotopic pregnancy in the second trimester, at 20 weeks of gestation [22].

There are few reports of heterotopic pregnancy that are diagnosed at term [4, 6, 15, 23]. In one study out of 112 heterotopic pregnancies after ovulation induction and assisted reproductive technologies 7 were diagnosed in the second trimester between 14 and 26 weeks [3]. Another case of late diagnosis was mentioned in the Danish survey which was diagnosed at 38 weeks during cesarean section [23]. A similar case of a full-term heterotopic pregnancy with a positive birth outcome was reported from Tanzania which was diagnosed on the 4^th^ postpartum day during laparotomy [4]. Another unique case reported by Kigbu JH, et al. in 2009 showed that a combined intrauterine and abdominal pregnancies was diagnosed at 38 weeks during elective Caesarean section for twin gestation with positive birth outcome [6]. Maciel N, et al., 2017 also reported a case of advanced abdominal pregnancy in a spontaneous heterotopic pregnancy, in which the intrauterine pregnancy was carried to term with a favorable outcome and the abdominal pregnancy was complicated by fetal demise [15].

The chance for both fetuses to reach term and survive the neonatal period is very low in twin heterotopic pregnancy [4]. According to a review of 11 cases done by Dubinsky, et al., 1996, on fetal survival in third trimester abdominal pregnancy revealed that four fetuses survived had complete placental attachment to the uterus but 6 out of 11 fetuses that died before delivery and in the early neonatal period had complete mesenteric placental attachment and partial placental attachment to the uterus [24]. Therefore, the site of placental attachment in peritoneal pregnancy is a crucial factor for fetal survival. In our case the placenta was attached to the mesentery, left broad ligament and the posterior uterine wall. Its partial attachment to the uterine wall may be the factor for its survival.

Therefore, based on our literature review a spontaneous heterotopic pregnancy which progress to term with both the IUP and the EUP survive up to the end of the neonatal period is very rare which makes our case exceptionally unique.

When we see events that lead to the diagnosis of heterotopic pregnancy, according to a literature review from 1971 to 1993 done by Tal et al., out of 111 reports in 64 women (58.9%), the heterotopic pregnancy was diagnosed during laparotomy or laparoscopy [3]. Sonographic detection of an extrauterine gestational sac with or without a fetal pole along with an IUP led to a definitive final diagnosis in 46 patients (41.1%) [3]. Since our patient has no ANC follow-up, the possibility of early diagnosis was missed. She was presented during the advanced stage of labor, so ultrasound examination was not done before delivery of the IUP. Therefore, the diagnosis of heterotopic pregnancy in our case was done on the immediate postpartum day during laparotomy.

Abdominal pregnancy is an alarming obstetric phenomenon [3]. Strictly defined, abdominal pregnancy is implantation in the peritoneal cavity exclusive of tubal, ovarian, or intraligamentary implantations [1]. Although a zygote can traverse the tube and implant primarily in the peritoneal cavity, most abdominal pregnancies are thought to follow early tubal rupture or abortion [1]. In our case, since the anatomy of the left adnexa was distorted, it was difficult to identify signs of early tubal rupture. Therefore, it is difficult to tell whether it is a primary or secondary peritoneal implantation. Abdominal pregnancies account for 1% of ectopic gestations [15]. When abdominal pregnancy is advanced, it has been associated with a maternal mortality of 12% and perinatal mortality of 72% [25]. Intrauterine growth restriction IUGR [26] and fetal malformations [3] are also common in advanced abdominal pregnancies which is consistent with our case.

The most frequent site of EP implantation in heterotopic pregnancy is the tube (89.2%) and abdominal heterotopic is one of the rarest types; Tal et al. reported that out of 139 heterotopic pregnancies which are conceived by ART, 3 were abdominal [3]. Oancea et al. also found out that out of 18 spontaneous heterotopic pregnancies reviewed only one was abdominal [22].

The diagnosis of abdominal-heterotopic pregnancy is a more complicated task [4]. The most important problem during ultrasound examination is that the sonographer has to be aware of the possibility of advanced abdominal pregnancy [4]. The identification of the uterus and the fetal head outside the uterine cavity may be diagnostic [3]. Fetal malpresentation as a transverse lie, the identification of an oligohydramnios [27, 28] and malformations [3] should, especially when occurring in combination, arouse suspicion. In equivocal cases, MRI can help establish diagnosis, localize the placenta’s implantation site, and in planning surgery [14].

These are consistent with our sonographic findings except the malformation was not identified. Otherwise, there was an empty uterus, and the fetal pole was in a transverse lie outside the uterus posteriorly. There was also oligohydramnios with a positive fetal heartbeat. Therefore, the diagnosis of abdominal heterotopic pregnancy was suspected by ultrasound examination and confirmed by laparotomy.

The most important issue in managing advanced abdominal pregnancy is the placental management [26]. The massive hemorrhage that often occurs with surgery is related to the lack of constriction of the blood vessels after placental separation [29]. Since the major cause of maternal death during surgery is related with an uncontrollable bleeding from placental separation site, determination of the placenta's implantation site is very crucial before its removal [26]. Some authors recommend that leaving the placenta in situ, with or without methotrexate administration to facilitate its reabsorption when the detachment of placenta is not feasible or safe [29]. Although it minimizes hemorrhage, this approach puts the patient at risk of necrosis, pelvic abscess, and wound dehiscence [29]. In a review of 139 reported heterotopic pregnancies two heterotopic pregnancies in which the EP was abdominal, removal of the gestational sac and placenta was accomplished easily, but in another case reported, during laparotomy, it was found that the feeder vessel to the placenta was the ovarian artery and salpingo-oophorectomy was performed [3]. In a case reported by Maciel et al., since the amniotic sac and placenta were clearly individualized, with no invasion of the pelvic sidewall, bowel, or mesentery, the excision of the mass was successfully achieved by left adnexectomy [15]. In the case of advanced abdominal pregnancy, with a live fetus reported by Hailu et al., they prefer to remove the placenta and they managed the bleeding by packing the area for 24 h [26]. In another case of a full-term abdominal pregnancy with isthmic tubal implantation of the placenta, it was removed by salpingectomy without any attempt to detach it from the tube [30]. The removal of the placenta is considered when it is safe and with a low risk of hemorrhage [4]. Hence, in our case, the placenta was spontaneously delivered and there was no complications encountered.

## Conclusions

This is a rare case of spontaneous heterotopic pregnancy with advanced abdominal ectopic in which both the intrauterine and the extra-uterine pregnancies survive. This case was also diagnosed and managed in a rural district hospital by mid-level professionals (non-physician surgeons). Our patient had two home deliveries and has no ANC follow-up during the current pregnancy. She has a low socioeconomic status and she can’t afford referral to a higher institution for better management for both herself and the low birth weight baby. Based on the findings on this case and our literature review, the following conclusions can be made.

Every health professional should bear in mind that intrauterine and extrauterine pregnancy may happen simultaneously and it can progress to term without any symptoms. Therefore, a high degree of suspicion is needed when we encounter abdominal mass after delivery of the IUP. Abdominal ectopic is a grave obstetric condition that needs early diagnosis and prompt management. Ultrasound is the diagnostic method of choice but the existence of an IUP cannot rule out ectopic pregnancy, therefore, adnexa should be routinely examined during the first-trimester scan. The life-threatening complication of abdominal ectopic pregnancy is bleeding from the detached placental site. Therefore, the decision to remove the placenta should be individualized.

## Data Availability

The datasets created during the study are available from the corresponding author on reasonable request.

## Abbreviations

ANC: Antenatal care
ART: Assisted reproductive technology
IUGR: Intrauterine growth restriction
IUP: Intrauterine pregnancy
EP: Ectopic pregnancy
MRI: Magnetic resonance imaging
CT scan: Computed tomography

## References

[1] Cunningham  FG, Leveno  KJ, Catherine  Y, Dashe  JS, Barbara  L (2014). Williams’s Obstetrics.

[2] Hassani KI, Bouazzaoui AE, Khatouf M, Mazaz K (2010). Heterotopic pregnancy: A diagnosis we should suspect more often. J Emerg Trauma Shock.

[3] Tal J, Haddad S, Gordon N, Timor-Tritsch I (1996). Heterotopic pregnancy after ovulation induction and assisted reproductive technologies: a literature review from 1971 to 1993. Fertil Steril.

[4] Ludwig M, Kaisi M, Bauer O, Diedrich K (1999). The forgotten child: a case of heterotopic, intra-abdominal and intrauterine pregnancy carried to term. Hum Reprod.

[5] Maaita ME, Murad N, Dabbas M (1999). Advanced heterotopic pregnancy. J Obstet Gynaecol.

[6] Kigbu JH, Sagay AS, Chingle PM (2009). Heterotopic pregnancy at term masquerading as intrauterine twin gestation, a case report. Niger J Clin Pract.

[7] Russman C, MGruner C, Jiang X, Schnatz PF. Spontaneous Heterotopic Pregnancy: A Case Report. Gynecol Obstet (Sunnyvale). 2015;5:318. 10.4172/2161-0932.1000318.

[8] McDonald J, Sebrina DP (2002). Abdominal Pain in the adolescent female. Clin Pediatr Emerg Med.

[9] Wang PH, Chao HT, Tseng JY, Yang TS, Chang SP (1998). Laparoscopic surgery for heterotopic pregnancies: a case report and a brief review. Obstetrics & Gynecology and Reproductive Biology.

[10] Berger MJ, Taymor ML (1972). Simultaneous intrauterine and tubal pregnancies following ovulation induction. Am J Obstet Gynecol.

[11] Reece EA, Petrie RH, Sirmans MF, Finster M, Todd WD (1983). Combined intrauterine and extra-uterine gestations: a review. Am J Obstet Gynecol.

[12] Chen KH, Chen LR (2014). Rupturing heterotopic pregnancy mimicking acute appendicitis. Taiwan J Obstet Gynecol.

[13] Spandana JC, Kanakannavar SS, Umashankar KM, Manuja N (2017). Spontaneous heterotopic pregnancy with tubal rupture. Int J Reprod Contracept Obstet Gynecol.

[14] Ali T, Tawab MA, Ghaffar ElHariri MAA, Ayad A (2020). Heterotopic pregnancy a case report. Egyptian Journal of Radiology and Nuclear Medicine.

[15] Maciel  N, Lima  AF, Cruz  R, Ponte  C (2017). Advanced abdominal pregnancy in a spontaneous heterotopic pregnancy. BMJ Case Rep.

[16] Kwon  YS, Lee  SH, Im KS, Ro JH (2015). Laparoscopic Management of Heterotopic Interstitial Pregnancy with Subsequent Term Delivery. Int J Fertil Steril.

[17] Porreco RP, Burke MS, Parker DW (1990). Selective embryocide in the nonsurgical management of combined intrauterine-extra uterine pregnancy. Obstet Gynecol..

[18] Kajdy A, Muzyka-Placzyńska K, Muzyka-Placzyńska D (2021). A unique case of diagnosis of a heterotopic pregnancy at 26 weeks–case report and literature review. BMC Pregnancy and Childbirth.

[19] Bright DA, Gaupp FB (1990). Heterotopic pregnancy: a reevaluation. J Am Board Fam Pract.

[20] Perkins KM, Boulet SL, Kissin  DM, Jamieson DJ, National ART, Surveillance (NASS) Group (2015). Risk of ectopic pregnancy associated with assisted reproductive technology in the United States, 2001–2011. Obstet Gynecol.

[21] Barrenetxea  G, Barinaga Rementeria L, Lopez de Larruzea A, Agirregoikoa  JA, Mandiola  M, Carbonero  K (2007). Heterotopic pregnancy: two cases and a comparative review. Fertil Steril.

[22] Oancea M, Ciortea R, Diculescu D, Poienar A, Grigore M, Lupean RA, Nicula R, Chira D, Strilciuc S, Mihu D (2020). Spontaneous Heterotopic Pregnancy with Unaffected Intrauterine Pregnancy: Systematic Review of Clinical Outcomes. Medicina.

[23] Svare J, Norup P, Grove Thomsen S, Hornnes P, Maigaard S, Helm P (1993). Heterotopic pregnancies after in-vitro fertilization and embryo transfer-a Danish survey. Hum Reprod.

[24] Dubinsky  TJ, Guerra  F, Gormaz  G, Maklad  N (1996). Fetal survival in abdominal pregnancy: a review of 11 cases. J Clin Ultrasound.

[25] Nkusu Nunyalulendho D, Einterz  EM (2008). Advanced abdominal pregnancy: case report and review of 163 cases reported since 1946. Rural Remote Health.

[26] Hailu FG, Yihunie GT, Essa AA, Tsega WK (2017). Advanced abdominal pregnancy, with live fetus and severe preeclampsia, case report. BMC Pregnancy Childbirth.

[27] Cartwright PS, Brown JE, Davis RJ (1986). Advanced abdominal pregnancy associated with fetal pulmonary hypoplasia: report of a case. Am J Obstet Gynecol.

[28] Moessinger AC (1986). Fetal lung growth in experimental utero abdominal pregnancy. Obstet Gynecol.

[29] Nassali MN, Benti TM, Bandani-Ntsabele M (2016). A case report of an asymptomatic late term abdominal pregnancy with a live birth at 41 weeks of gestation. BMC Res Notes.

[30] Sib SR, Ouédraogo  I, Sanogo  M, Kiemtoré  S, Sawadogo YA, Zamané  H, Bonané  B (2018). A full term abdominal pregnancy with an isthmic tubal implantation of the placenta. BMC Pregnancy and Childbirth.

